# Hydroxysafflor Yellow A Protects Neurons From Excitotoxic Death through Inhibition of NMDARs

**DOI:** 10.1177/1759091416642345

**Published:** 2016-04-09

**Authors:** Xingtao Wang, Zhiyuan Ma, Zhongxiao Fu, Su Gao, Liu Yang, Yan Jin, Hui Sun, Chaoyun Wang, Weiming Fan, Lin Chen, Qing-Yin Zheng, Guoqiang Bi, Chun-Lei Ma

**Affiliations:** 1 Department of Physiology, Binzhou Medical University, Yantai, Shandong, China; 2 “Brain stroke” Key Lab of Shandong Health Administration Institute, Binzhou Medical University, Yantai, Shandong, China; 3 Department of Internal Neurology, Affiliated Hospital of Binzhou Medical University, Binzhou, Shandong, China; 4 School of Public Economics and Administration, Shanghai University of Finance and Economics, Shanghai, China; 5 CAS Key Laboratory of Brain Function and Diseases, School of Life Science, University of Science and Technology of China, Hefei, Anhui, China; 6 Department of Pharmacology, Binzhou Medical University, Yantai, Shandong, China

**Keywords:** hydroxysafflor yellow A, Ca^2+^ imaging, NMDA receptor, oxygen-glucose deprivation, whole-cell patch-clamp recording

## Abstract

Excessive glutamate release causes overactivation of N-methyl d-aspartate receptors (NMDARs), leading to excitatory neuronal damage in cerebral ischemia. Hydroxysafflor yellow A (HSYA), a compound extracted from *Carthamus tinctorius* L., has been reported to exert a neuroprotective effect in many pathological conditions, including brain ischemia. However, the underlying mechanism of HSYA's effect on neurons remains elusive. In the present study, we conducted experiments using patch-clamp recording of mouse hippocampal slices. In addition, we performed Ca^2+^ imaging, Western blots, as well as mitochondrial-targeted circularly permuted yellow fluorescent protein transfection into cultured hippocampal neurons in order to decipher the physiological mechanism underlying HSYA's neuroprotective effect.

Through the electrophysiology experiments, we found that HSYA inhibited NMDAR-mediated excitatory postsynaptic currents without affecting α-amino-3-hydroxy-5-methyl-4-isoxazolepropionic acid receptor and γ-aminobutyric acid A-type receptor-mediated currents. This inhibitory effect of HSYA on NMDARs was concentration dependent. HSYA did not show any preferential inhibition of either N-methyl d-aspartate receptor subtype 2A- or N-methyl d-aspartate receptor subtype 2B- subunit-containing NMDARs. Additionally, HSYA exhibits a facilitatory effect on paired NMDAR-mediated excitatory postsynaptic currents. Furthermore, HSYA reduced the magnitude of NMDAR-mediated membrane depolarization currents evoked by oxygen-glucose deprivation, and suppressed oxygen-glucose deprivation–induced and NMDAR-dependent ischemic long-term potentiation, which is believed to cause severe reperfusion damage after ischemia. Through the molecular biology experiments, we found that HSYA inhibited the NMDA-induced and NMDAR-mediated intracellular Ca^2+^ concentration increase in hippocampal cultures, reduced apoptotic and necrotic cell deaths, and prevented mitochondrial damage. Together, our data demonstrate for the first time that HSYA protects hippocampal neurons from excitotoxic damage through the inhibition of NMDARs. This novel finding indicates that HSYA may be a promising pharmacological candidate for the treatment of brain ischemia.

## Introduction

Glutamate is a major excitatory neurotransmitter in the mammalian central nervous system (CNS). The N-methyl d-aspartate (NMDA) type of glutamate receptors plays important roles in mediating glutamatergic synaptic transmission, neuronal growth, neurodevelopment, learning, and memory. N-methyl d-aspartate receptors (NMDARs) also play a pivotal role in facilitating neuronal death under pathological conditions such as brain ischemia (Martin and Wang, 2010).

Excessive glutamate release in the ischemic brain induces a localized increase in glutamate concentration that overactivates NMDARs, thereby causing excitatory neuronal death (Du et al., 1997; Mehta et al., 2007). Previous studies have indicated that the local concentration of glutamate may remain elevated for more than 3 days after an ischemic stroke (Dohmen et al., 2003). Moreover, many neurons in the peri-ischemic or penumbral regions show morphological and biochemical features of apoptotic cell death, a process mediated by NMDAR overactivation; this phenomenon can last for hours or even days (Calabresi et al., 2003; Li et al., 2012). All these studies further propose that the administration of NMDAR antagonists could prevent neuronal death and confer neuroprotective effects after ischemic strokes. Consistent with this notion, NMDAR antagonists have demonstrated obvious neuroprotective effects *in vivo* and *in vitro* (Manabe et al., 1993; Planells-Cases et al., 2006). However, subsequent clinical trials involving the therapeutic use of NMDAR antagonists in brain ischemia failed due to the fact that the NMDARs play important roles in maintaining normal synaptic transmission and neuronal growth, as well as regulating neuronal survival and recovery following brain ischemia (Birmingham, 2002; Lipton, 2006).

Interestingly, memantine, a low-affinity NMDAR antagonist, has been shown to be effective in treating moderate to severe Alzheimer's disease without exhibiting significant clinical side effects (Chen and Lipton, 2006). One possible explanation is that memantine preferentially blocks excessive NMDAR activity but maintains a certain level of background activity which is needed for normal synaptic transmission (Chen and Lipton, 2006). Therefore, we attempt to find a pharmacological agent possessing a similar mechanism of action as memantine, which could make it a more suitable agent in the clinical treatment of ischemic stroke.

Hydroxysafflor yellow A (HSYA) is the main chemical component of safflower yellow pigments. Previous studies have shown that HSYA inhibits platelet aggregation (Zang et al., 2002), can cross the brain–blood barrier (Liu et al., 2013), and exhibits neuroprotective effects after permanent middle cerebral artery occlusion in rats (Zhu et al., 2003; Wei et al., 2005). Many studies have also indicated that HSYA protects the brain against permanent middle cerebral artery occlusion and cerebral ischemia/reperfusion injury by reducing the infarct volume in the ipsilateral hemisphere (Zhu et al., 2003), suppressing inflammatory responses following focal ischemic reperfusion (Ye and Gao, 2008), and exerting an antioxidant action (Wei et al., 2005). In addition, Yang and colleagues (2010) reported that the neuroprotective effect of HSYA was partially attributed to the downregulation of N-methyl d-aspartate receptor subtype 2B (NR2B)-containing NMDAR expression in cultured cortical neurons. Although studies have shown that HSYA protects neurons against glutamatergic excitotoxicity, the underlying mechanism of this action needs to be clarified (Zhu et al., 2003; Tian et al., 2004; Liu et al., 2013). In the present study, we demonstrate that HSYA protects neurons from ischemic damage through the inhibition of NMDARs.

## Materials and Methods

### Brain Slice Preparation

All animal care and experimental procedures complied with local and internal guidelines on ethical use of animals and were approved by the University's animal welfare committee. In electrophysiology experiments, each group of data was collected from at least six mice. Coronal brain slices containing the hippocampus were obtained as described previously (Imamura et al., 2008; Basu et al., 2009; Bakkar et al., 2011; Amini et al., 2013; Gao et al., 2015). In brief, prior to decapitation, postnatal 4 to 6 weeks C57BL/6 mice were anesthetized with ether. The brain was quickly removed and placed in a CO_2_-balanced, oxygenated (95% O_2_ and 5% CO_2_) normal artificial cerebral spinal fluid (ACSF) at 4℃ containing (in mM): 130 NaCl, 3 KCl, 1.3 MgCl_2_, 20 NaHCO_3_, 1.2 KH_2_PO_4_, 2.4 CaCl_2_, 3 HEPES (4-(2-hydroxyethyl)-1-piperazineethanesulfonic acid), and 10 glucose. The osmolarity of the ACSF was adjusted to 300 to 310 mOsm, and the pH was adjusted to 7.2 to 7.4. Coronal brain slices (300 µm) were obtained using a vibrating microtome (VT 1000S; Leica, Germany) at 4℃. The slices were then transferred to a chamber containing oxygenated normal ACSF and maintained at room temperature for at least 1 h prior to patch-clamp recordings.

### Patch-Clamp Recordings

Whole-cell patch-clamp recordings of hippocampal CA1 pyramidal cells (CA1 PCs) were obtained with a Multiclamp 700B amplifier (Molecular Devices) under visual control using differential interference contrast and infrared video microscopy (Leica, Germany). The recording electrode had a resistance of 4 to 6 MΩ, and the access resistance was 10 to 30 MΩ. During the recordings, if the series resistance or the cell capacitance deviated by >20% from initial values, the cells were excluded from analysis. Data were collected with pClamp10.2 software, and offline analysis was done using pClampfit10.2 (Molecular Devices Orleans, USA).

For whole-cell voltage-clamp recordings on CA1 PCs of mouse hippocampal slices, the recording electrode was filled with an intracellular solution containing (in mM): 130 Cs-methanesulphonate, 10 HEPES, 10 CsCl, 2 MgCl_2_, 2 adenosine triphosphate magnesium, 1 QX-314 (lidocaine N-ethyl bromide), and 2 EGTA. For current-clamp recordings, the recording electrode was filled with an intracellular solution containing (in mM): 130 K-gluconate, 10 KCl, 10 HEPES, 2 MgCl_2_, 1 EGTA, and 2 adenosine triphosphate magnesium. The osmolarity was adjusted to 280 to 290 mOsm and the pH was adjusted to 7.2 to 7.4. For recording miniature excitatory postsynaptic currents (mEPSCs), we used low magnesium ion (Mg^2+^) ACSF containing (in mM): 130 NaCl, 3 KCl, 0.1 MgCl_2_, 20 NaHCO_3_, 1.2 KH_2_PO_4_, 2.9 CaCl_2_, 3 HEPES, and 10 glucose, to which was added tetrodotoxin (TTX) (1 μM), picrotoxin (PTX, 100 μM), and strychnine (1 μM) in order to block voltage-dependent Na^+^ channels, γ-aminobutyric acid A-type receptors (GABA_A_ receptors), and glycine receptors, respectively.

For recording postsynaptic currents, a bipolar microelectrode was placed on the Schaffer collaterals (SCs) in the stratum radiatum, delivering 100 μs pulse currents at the stimulation frequency of 0.06 to 0.08 Hz. For recording NMDAR-mediated EPSCs, 6-cyano-7-nitroquinoxaline-2,3-dione (CNQX; 5 μM), PTX (100 μM), and strychnine (1 μM) were included in the low Mg^2+^ ACSF to block α-amino-3-hydroxy-5-methyl-4-isoxazolepropionic acid receptors (AMPARs), GABA_A_, and glycine receptors, respectively. The cell membrane potential (in millivolts) was clamped at −30mV to maximize the current response. For recording AMPAR-mediated EPSCs, PTX (100 μM) and strychnine (1 μM) were included in the normal ACSF, and the cell membrane potential was clamped at −65 mV. The ratio of EPSCs of AMPA/NMDA was calculated by dividing the peak AMPAR current obtained at −65mV over the NMDAR current obtained at +40mV. The peak NMDAR current was measured with V-holding of +40 mV, at a time point 3 times the AMPAR EPSC's decay time constant (Beique et al., 2006). For recording GABA_A_ receptor-mediated inhibitory postsynaptic currents, CNQX (5 μM), 2-amino-5-phosphonopentanoic acid (APV; 100 μM), and strychnine (1 μM) were included in the normal ACSF at the V-holding of −30 mV.

Because Ro25-6981 is the NR2B selective blocker which has a 5,000-fold affinity for NR2B than for N-methyl d-aspartate receptor subtype 2A (NR2A; Fischer et al., 1997); therefore, for recording NR2A-containing NMDAR EPSCs, we bath-applied 1 μM of Ro25-6981 (SML0495, Sigma–Aldrich, USA) after pharmacologically isolating NMDAR EPSCs. For evaluating the contribution of NMDAR activity to the intrinsic membrane property of neurons, CNQX (5 μM), PTX (100 μM), and strychnine (1 μM) were included in the normal ACSF in current-clamp experiments. Action potentials were elicited by injecting hyperpolarizing and depolarizing currents through the recording electrode and measuring the ensuing membrane voltage (the injected currents ranged from −120 pA to +120 pA with a step of 20 pA). The membrane voltage was then plotted as the function of the injected current.

For recording NMDAR-mediated anoxic depolarizing currents, we used an oxygen-glucose deprivation (OGD) solution containing (in millimolars): 130 NaCl, 3 KCl, 0.1 MgCl_2_, 20 NaHCO_3_, 1.2 KH_2_PO_4_, 2.9 CaCl_2_, 3 HEPES, and 7 sucrose, while bubbling the solution with 95%N_2_/5%CO_2_. Iodoacetate (2 mM), cyanogen bromide (1 mM), and ascorbic acid (5 mM) were also added to the OGD solution to obtain a reproducible fast-onset ischemic response (Rossi et al., 2000). In addition, PTX (100 μM), strychnine (1 μM), CNQX (5 μM), and nifedipine (10 μM) were added to respectively block AMPARs, GABA_A_ receptors, glycine receptors, and voltage-gated calcium channels. Before recording the NMDAR-mediated and OGD-induced cell membrane depolarization currents, a 5-min baseline current in low Mg^2+^ ACSF was first recorded. The slices were then perfused in OGD solution for 6 min before being returned to low Mg^2+^ ACSF. For recording OGD-induced ischemic long-term potentiation (i-LTP), the perfusing solution was switched from a low Mg^2+^ ACSF containing PTX (100 μM) and strychnine (1 μM) to the OGD solution for 3 min, then switched back to low Mg^2+^ ACSF (Arcangeli et al., 2013).

### Primary Culture of Hippocampal Neurons

Dissociated cultures of hippocampal neurons were prepared from postnatal 0- to 1-day mice. The tissue was isolated by a standard enzyme treatment protocol. Briefly, shredded tissue was treated with 0.125% trypsin (T1426, Sigma–Aldrich, USA) and incubated for 25 min at 37℃. After centrifuging the mixture, the supernatant was discarded. The neurons were plated onto coverslips coated with poly-l-lysine (D0301, Sigma–Aldrich, USA) and then grown in Dulbecco's Modified Eagle Medium/Nutrient Mixture F-12 and 10% fetal calf serum (21103-049, Gibco, USA) for the first 24 h. Neuronal cultures were kept at 37℃ in a 5% CO_2_ humidified atmosphere. The culture medium was replaced by neurobasal containing 2% B27 on the second day. After that, the culture medium was replaced every 3 days. Glial growth was suppressed by addition of 5-fluoro-2-deoxyuridine (10 μM) and uridine (10 μM) (Sigma–Aldrich, USA). Mature neurons (12–16 DIV) were then used for our experiments. To induce neuronal injury, hippocampal cultures were washed three times in Mg^2+^-free extracellular solution (ECS) containing (in millimolars): 140 NaCl, 5 KCl, 2 CaCl_2_, 20 HEPES, and 3 glucose, with a pH of 7.4 and an osmolarity of 310 to 320 mOsm.

### Ca^2+^ Imaging

Cultured hippocampal neurons grown on 8 × 8 mm glass coverslips were transferred to 12 well plates and washed twice with normal ECS; they were then incubated in the dark in normal ECS with Fluo-4 AM (4 μM; F312, Dojindo Laboratories, Japan) for 60 min at 37℃. Following incubation, the cultures were first washed in ECS and equilibrated for 30 min at room temperature, and then washed in modified ECS (Mg^2+^-free, 3 mM glucose, 2.9 mM Ca^2+^). After this, coverslips were transferred into a container on an inverted confocal microscope (LEICA TCS SPE, Germany) and incubated with modified ECS for 15 min at room temperature; the modified ECS contained 1,2,3,4-tetrahydro-6-nitro-2,3-dioxobenzo[f]quinoxaline-7-sulfonamide (5 μM), bicuculline (10 μM), strychnine (1 μM), and nifedepine (10 μM) to block AMPARs, GABA_A_ receptors, glycine receptors, and VGCCs, respectively. Before scanning, the container was placed onto the stage of a confocal microscope equipped with a 63× oil-immersion objective lens and set in xyt mode with a laser wavelength of Ex = 488 nm and Em = 530 nm. The resolution of frames scanned was set to 512 × 512 pixels.

Five-minute baseline images followed by 30-min experimental treatment images were taken. The intensity of fluorescence was analyzed using the dynamic intensity analysis module of Leica Application Suite AF. To normalize variations in Ca^2+^ loading, we used the relative fluorescence intensity Δ*F_t_*/F_0_ to express the changes in intracellular Ca^2+^ concentration (i.e., folds of Ca^2+^ increase). *F_t_* is the averaged fluorescence intensity of ROI from each frame at a given time, and *F*_0_ is the averaged 5-min baseline fluorescence intensity from the same region of interest (ROI) immediately prior to experimental treatments. Background fluorescence (from a region within the same field lacking cells) was subtracted from ROI averages (Feldman et al., 2008).

### Cell Viability Assay

Apoptotic neuronal death was measured by visualizing neurons stained with Hoechst-33342 (C1022, Beyotime Institute of Biotechnology, China). To stain apoptotic neurons, Hoechst-33342 (5 µg/ml) was added to the culture medium 24 h after experimental treatment; cultures were then incubated at 37℃ for 25 min. Images were taken with a fluorescence microscope (IX71SIF-3, Olympus, Japan). Cells with condensed or fragmented chromatin morphology were considered to be apoptotic. These observations were quantified by double-blind counting the number of apoptotic versus total neurons in each visual field, and expressing the results as a percentage ratio. Necrotic cell death was quantified by measuring the amount of lactate dehydrogenase (LDH) released into the culture medium 24 h after experimental treatments using a Cyto Tox 96 assay kit (Promega, Madison, WI). The absorbance readings were measured using a microplate reader (MULTISKAN MK3, Thermo Scientific, USA).

### Western Blot

Hippocampal cultures treated with NMDA for 30 min at room temperature were lysed in cell lysis buffer (20 mM Tris pH 7.5, 150 mM NaCl, 1% Triton X-100, 2.5 mM sodium pyrophosphate, 1 mM EDTA, 1% Na_3_VO_4_, 0.5 µg/ml leupeptin, and 1 mM phenylmethanesulfonyl fluoride). Protein samples (30 µg) were run in sodium dodecyl sulfate–polyacrylamide gels (12%) for 1.5 h using Tris-glycine running buffer. The gels were then transferred onto a polyvinylidene fluoride membrane in transfer buffer containing 25 mM Tris-base, 0.2 M glycine, and 20% (v/v) methanol for 1 h at 70 V. The polyvinylidene fluoride membrane was blocked with 5% nonfat milk in 1× tris-buffered saline, 0.1% Tween-20 at 25℃ for 1 h; it was subsequently incubated overnight at 4℃ with primary antibodies for cleaved caspase-3 at Asp175 (rabbit anti-mouse, 1:1,000, Cell Signaling Technology, USA) and β-tubulin (rabbit anti-mouse, 1:1,000, Cell Signaling Technology, USA). After washing with tris-buffered saline ansd Tween-20, the membrane was incubated with a horseradish peroxidase–conjugated secondary antibody (Goat anti-rabbit IgG, ZSGB-BIO, China) for 1 h, and protein signals were detected with a chemiluminescence system (Clinx Science Instruments Shanghai, China). Quantification of protein levels was achieved by densitometry analysis using Image J software, and expressed as the ratio of the values of the detected protein band versus the β-tubulin band.

### Transfection of Mitochondrial-Targeted Circularly Permuted Yellow Fluorescent Protein and Confocal Imaging

Dissociated neurons from E18 hippocampus of Sprague-Dawley rats were plated onto poly-lysine–coated 10 mm glass coverslips and cultured in Dulbecco's Modified Eagle Medium/Nutrient Mixture F-12 and 10% fetal calf serum (21103-049, Gibco, USA) at 37ºC in a 5% CO_2_ humidified atmosphere. After 24 h, the culture medium was changed to neurobasal containing 2% B27. The neurobasal medium was replaced every 3 days thereafter. At 8 to 9 DIV, neuronal cultures were transfected with plasmids carrying the mitochondrial-targeted circularly permuted YFP (mt-cpYFP) using the calcium phosphate precipitation method. These transfected cultures were allowed to grow for another 7 days before use in experiments.

During the experiments, coverslips were transferred into a chamber on the stage of a confocal microscope (Zeiss LSM 710, Germany) and perfused with Mg^2+^-free ACSF containing TTX (1 μM), CNQX (5 μM), bicuculline (10 μM), and strychnine (1 μM) to block voltage-dependent Na^+^ channels, AMPARs, GABA_A_ receptors, and glycine receptors, respectively. Images were taken with a confocal microscope equipped with a 20× water-immersion objective lens. Samples was excited with a 488-nm laser and detected at 530-nm emission. Time-lapse *x,y* images were acquired using 2s/frame at an image resolution of 512 × 512 and scanning interval of 1 min. The changes in relative fluorescence intensities were expressed as Δ*F_t_*/*F*_0_.

To assess for NMDAR-mediated mitochondrial damage, confocal images were obtained at baseline, during a 5-min bath application of NMDA (100 μM NMDA and 10 μM glycine), and after reperfusion of the culture with the control solution (this step mimics tissue reperfusion after brain ischemia). To observe HSYA's neuroprotective effect, neuronal cultures were preincubated in HSYA-containing ACSF, and kept in HSYA-containing perfusion solution during experiments.

### Statistical Analysis

Student's *t* tests (two tailed) and Kolmogorov–Smirnov (K–S) test were used to determine the statistical significance of the results. Analysis of variance (ANOVA) was used for comparing data from multiple groups, followed by post-hoc analysis for comparing data between two groups. Numerical results were expressed as mean ± *SE.* The statistic significance of the difference between values was set as a *p* value less than .05 (*i.e.,* *p* < .05).

## Results

### HSYA Inhibits Excitatory Postsynaptic Currents

**Figure 1.**
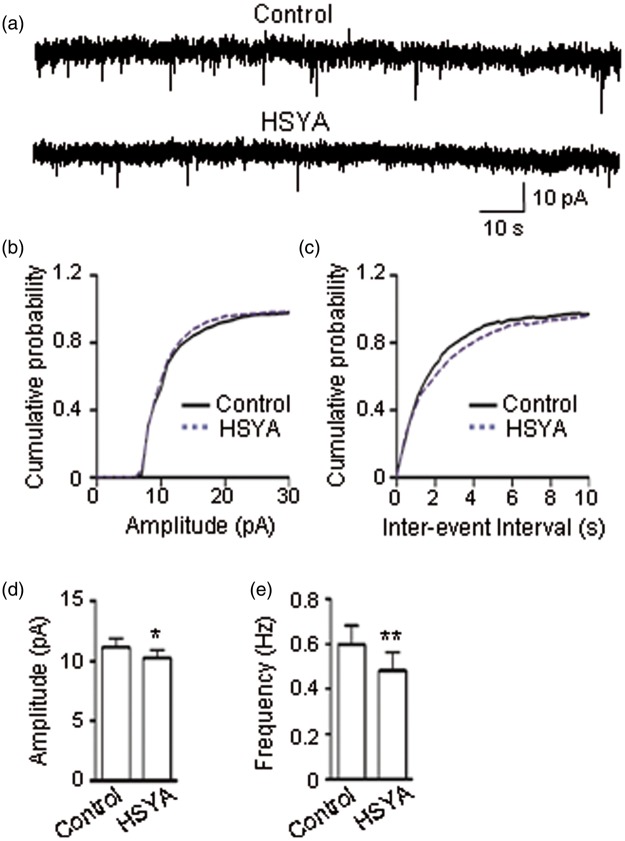
HSYA inhibits mEPSCs. (a) Consecutive mEPSCs recorded in CA1 PCs before (upper trace) and during (lower trace) the bath application of HSYA (100 μM) in mouse hippocampal slices. Traces represent 1 min recordings. (b–c) Cumulative probability plots of data from (a). HSYA reduced the amplitude (b; *n* = 10; *p <* .0001, K–S test) and increased the inter-event interval of mEPSCs (c; *n* = 10; *p <* .0001, K–S test). (d–e) Effects of HSYA on the mean amplitude and frequency of mEPSCs. HSYA reduced the mean amplitude (d; *n* = 10; *p =* .02, paired *t* test) and the mean frequency of mEPSCs (e; *n* = 10; *p* = .0015, paired *t* test). Data shown as mean ± SE. *p < .05, **p < .01 compared with control. HSYA = hydroxysafflor yellow A; K–S = Kolmogorov–Smirnov; mEPSC = miniature excitatory postsynaptic current; PC = pyramidal cell.

To explore HSYA's effect on glutamatergic synaptic transmission in mouse hippocampal slices, we conducted voltage-clamp experiments by holding the membrane potential of CA1 pyramidal neurons at −65 mV and recording the mEPSCs (see Methods section). We found that HSYA (100 μM) caused a significant reduction in the amplitude of mEPSCs (Figure 1a and b; *p* < .0001, K–S test). HSYA also caused an obvious reduction in the frequency of mEPSCs as reflected by the increase in interevent interval (Figure 1a and c; *p* < .0001, K–S test). The mean mEPSC amplitude of the control group was 11.15 ± 0.74 pA; after the addition of HSYA to the bath solution, the mean mEPSC amplitude was reduced to 10.26 ± 0.64 pA (Figure 1d; *p* = .018, paired *t* test). The mean frequency of mEPSCs of the control group was 0.60 ± 0.08 Hz; after HSYA addition, the frequency was markedly reduced to 0.48 ± 0.08 Hz (Figure 1e; *p* = .0015, paired *t* test). These data suggest that HSYA inhibits EPSCs.

### HSYA Inhibits Postsynaptic NMDAR activity

**Figure 2.**
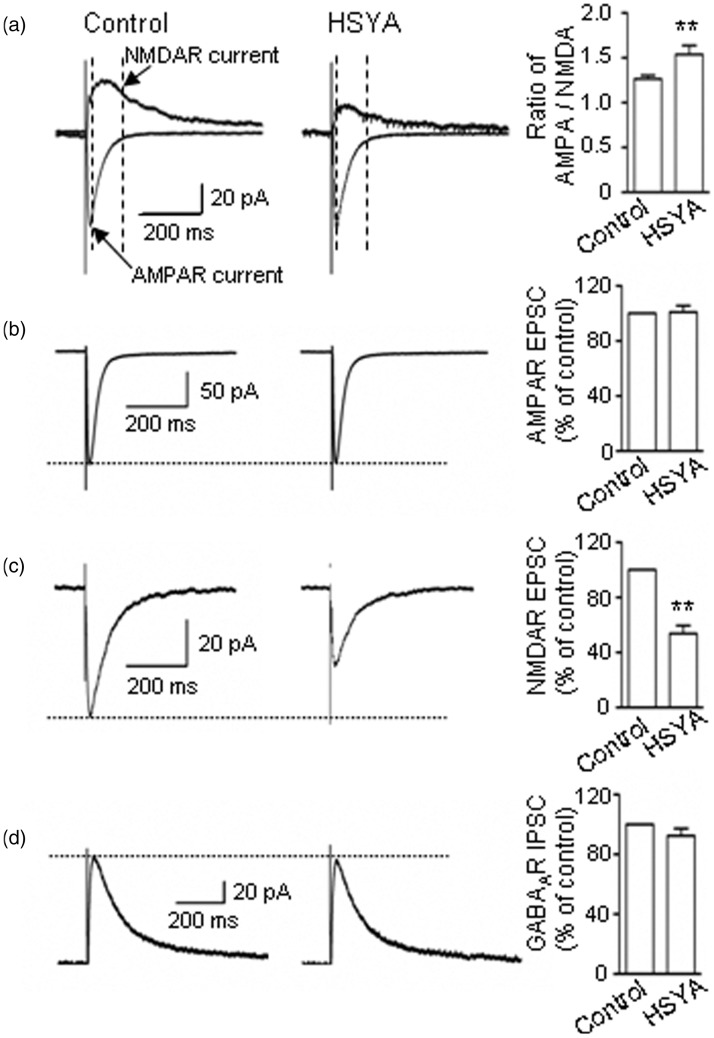
HSYA inhibits NMDAR-mediated EPSCs. (a) AMPAR- and NMDAR-mediated EPSCs were recorded at the holding potentials of −65 mV and +40 mV (left panel) in CA1 PCs of mouse hippocampal slices. At −65 mV, AMPAR-mediated EPSCs contributed the most to the peak current (as indicated by a pointed arrow; left panel), whereas at +40 mV, the peak current comes from both AMPAR- and NMDAR-mediated EPSCs. NMDAR EPSCs were measured at an indicated point (arrow). HSYA (100 μM) greatly reduced the NMDAR-mediated current (middle panel) and increased the ratio of AMPAR/NMDAR EPSCs (right panel; *n* = 7). Bar graphs showing the mean ratio (± *SE*) of AMPA/NMDA EPSC. **p < .01 compared with control, paired *t* test. (b) Representative traces (left and middle panels), and pooled data (right panel) showing that HSYA (100 μM) did not inhibit AMPAR EPSCs (*n* = 10). (c) Representative traces (left and middle panels), and pooled data (right panel) showing that the amplitude of NMDAR EPSCs was greatly reduced by HSYA (100 μM; *n* = 14). Bar graphs showing the mean NMDAR EPSC amplitudes (± *SE*). **p < .01 compared with control, paired *t* test. (d) Representative traces (left and middle panels) and pooled data (right panel) showing that HSYA (100 μM) did not inhibit GABA_A_ receptor-mediated IPSCs (*n* = 6). AMPAR = α-amino-3-hydroxy-5-methyl-4-isoxazolepropionic acid receptor; EPSC = excitatory postsynaptic current; GABA_A_ = γ-aminobutyric acid A-type; HSYA = hydroxysafflor yellow A; IPSC = inhibitory postsynaptic currents; NMDAR = N-methyl d-aspartate receptor; PC = pyramidal cell.

As the recorded mEPSCs contained both AMPAR- and NMDAR-mediated components, to further explore whether HSYA affects the AMPAR and/or the NMDAR component, we applied electrical stimulation to the SCs and recorded the EPSCs while holding the membrane voltage at either −65 mV or +40 mV (see Methods section). We found that the ratio of AMPAR/NMDAR currents was increased from 1.26 ± 0.04 (before HSYA application) to 1.54 ± 0.10 (after HSYA application; Figure 2a; *p* = .007, paired *t* test).

The increase in the AMPAR/NMDAR ratio might be due to either an increase in the amplitude of AMPAR EPSCs or a decrease in the amplitude of NMDAR EPSCs. To clarify the exact effects of HSYA on AMPAR and NMDAR activity, we pharmacologically isolated AMPAR EPSCs and NMDAR EPSCs, then tested HSYA's effect on each of them. The results demonstrated that HSYA markedly reduced the amplitude of NMDAR-mediated EPSCs to 53.6 ± 6 % that of control (Figure 2c; *p* = .0014, paired *t* test), but did not affect the amplitude of AMPAR EPSCs (100.93% ± 4.60% of control; Figure 2b; *p* = .90, paired *t* test).

We also investigated the effect of HSYA on GABA_A_ receptor-mediated IPSCs and found that the amplitude of GABA_A_-mediated IPSCs was not affected by HSYA application (96.67 ± 6.74 % of control; Figure 2d; *p* = .30, paired *t* test). Taken together, these results suggest that HSYA inhibits NMDAR activity but does not significantly affect the activity of AMPAR or GABA_A_ receptors.

**Figure 3.**
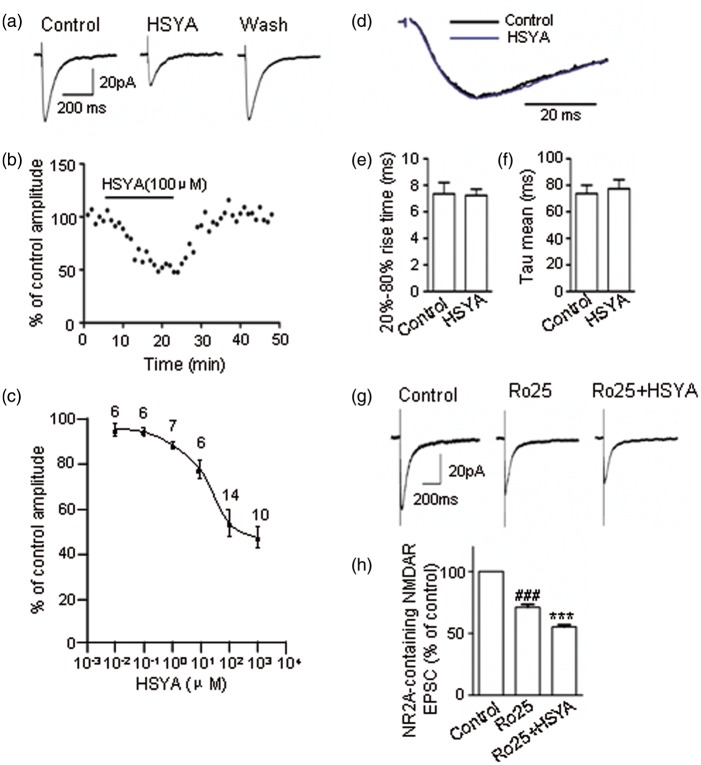
HSYA inhibits NMDAR EPSCs in a concentration-dependent manner. (a) Representative traces of pharmacologically isolated and electrically evoked NMDAR EPSCs in CA1 PCs, in the absence or presence of HSYA. The NMDAR EPSCs were reversibly inhibited by bath application of HSYA (100 μM). (b) The time course of HSYA-induced inhibition of NMDAR EPSCs. The inhibitory effect of HSYA on the amplitude of NMDAR EPSCs was time dependent. A plateau was reached at about 20 min following the bath application of HSYA. The amplitude of NMDAR EPSCs rapidly recovered to baseline level within about 6 min of HSYA's removal from the bath solution. (c) Concentration–response curve showing that HSYA partially inhibited NMDAR EPSCs. Even at relatively high concentrations, HSYA only reduced the amplitude of NMDAR EPSCs to 53.60 ± 6%–47 ± 4.85% of the control. The half-maximum inhibitory concentration of HSYA was 17.60 μM. (d) Normalized NMDAR EPSCs in the absence or presence of HSYA (100 μM). (e–f) Bar graphs showing the 20–80% rise time (e), and the weighted decay time constant (f). HSYA (100 μM) addition did not have a significant effect on the 20–80% rise time or the decay time constant (*n* = 12). (g) Representative traces of NMDAR EPSCs (left panel) and NR2A-containing NMDAR EPSCs in the absence (middle panel) or presence (right panel) of HSYA (100 μM). HSYA reduced the amplitude of NR2A-containing NMDAR EPSCs (*n* = 6). (h) Bar graphs showing the EPSC amplitude of the treatment group as a mean percentage (± *SE*) of control. ^###^*p* < .001 compared with control group, ***p < .001 compared with Ro25-6981 group, paired *t* test. EPSC = excitatory postsynaptic current; HSYA = hydroxysafflor yellow A; NMDAR = N-methyl d-aspartate receptor; PC = pyramidal cell.

To further examine the inhibitory efficacy of HSYA on NMDAR activity, we administrated different concentrations of HSYA to the bath solution. As shown in Figure 3a and b, HSYA reversibly inhibited NMDAR EPSCs; this inhibitory effect was concentration dependent in the range of 0.1 to 100 μM of HSYA. The half-maximum inhibitory concentration (IC50) was 17.60 μM (Figure 3c). Interestingly, HSYA did not appear to affect NMDAR channel kinetics (Figure 3d–f; *p* = .20, paired *t* test).

We also explored the subunit selectivity of the inhibitory effect of HSYA on NMDARs. The amplitude of NMDAR EPSC was reduced to 74.90 ± 2.25% of control when NR2B blocker Ro25-6981 (1 μM) was added to the bath solution for 20 min (Figure 3g and h; *p* = .000002, paired *t* test). Following this, when HSYA was added to the solution, the EPSC amplitude was further reduced to 77.60 ± 2.50 % of the Ro25-6981-treatment group (Figure 3g and h; *p* = .00004, paired *t* test).

### HSYA Suppresses Pre-Synaptic Glutamate Transmitter Release

**Figure 4.**
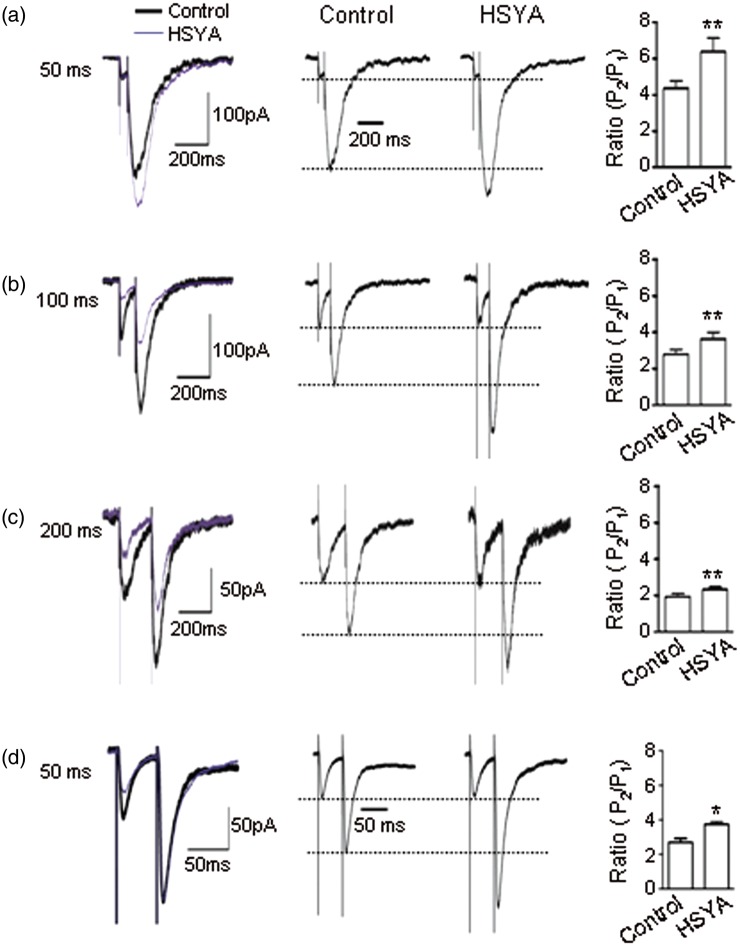
HSYA increases the ratios of both NMDAR- and AMPAR-mediated EPSC2/EPSC1. Sample traces of NMDAR-mediated EPSCs and AMPAR-mediated EPSCs evoked by paired-pulse stimulation. Representative actual and normalized traces of NMDAR-mediated EPSCs at the PP intervals of 50 ms, 100 ms, and 200 ms (a–c). The amplitude of the second NMDAR EPSCs (*P*2) was higher than the first (*P*1). After HSYA (100 μM) application, the amplitudes of *P*2 were further enhanced over *P*1 (50 ms, *n* = 7; 100 ms, *n* = 9; 200 ms, *n* = 12). (d) Representative actual and normalized traces of AMPAR-mediated EPSCs at the PP intervals of 50 ms. The amplitudes of the second AMPAR EPSCs (*P*2) were higher than the first (*P*1). After HSYA (100 μM) application, the amplitudes of *P*2 were further enhanced over *P*1 (*n* = 6). Bar graphs showing the mean (± *SE*) *P*2/*P*1 ratio, *p < .05, **p < .01 compared with control, paired *t* test. AMPAR = α-amino-3-hydroxy-5-methyl-4-isoxazolepropionic acid receptor; EPSC = excitatory postsynaptic current; HSYA = hydroxysafflor yellow A; NMDAR = N-methyl d-aspartate receptor.

To explore whether HSYA affects pre-synaptic glutamate transmitter release, we applied paired-pulse stimulation to SCs and recorded the NMDAR-mediated EPSCs. We measured the peak of the paired-pulse responses at the stimulus intervals of 50 ms, 100 ms, and 200 ms, and then calculated the ratios of *P*2/*P*1 (PP ratio [PPR]). We found that the PPR was increased at all stimulus intervals after the addition of HSYA (100 μM) to the bath solution (Figure 4a–c). At the stimulus interval of 50 ms, the ratio of *P*2/*P*1 was 4.40 ± 0.40. After bath application of HSYA, the PPR was markedly increased to 6.40 ± 0.70 (Figure 4a; *p* = .004, paired *t* test). At the stimulus interval of 100 ms, the PPR was 2.80 ± 0.30 before HSYA application, and 3.60 ± 0.40 after HSYA application (Figure 4b; *p* = .002, paired *t* test). At the stimulus interval of 200 ms, the PPR was 1.90 ± 0.30 before HSYA, increasing to 2.30 ± 0.10 after HSYA (Figure 4c; *p* = .006, paired *t* test).

We further investigated the PPR of AMPAR-mediated EPSCs by applying the paired-pulse at 50-ms stimulation intervals. We found that the PPR of AMPAR EPSCs was also increased after bath application of HSYA (Figure 4d; *p* = .03, paired *t* test).

As the release probability of pre-synaptic neurotransmitters was inversely correlated with the paired-pulse ratio (Yan and Weng, 2013), our data suggest that HSYA suppresses glutamate transmitter release in the pre-synaptic terminals of CA1 region of the mouse hippocampus.

**Figure 5.**
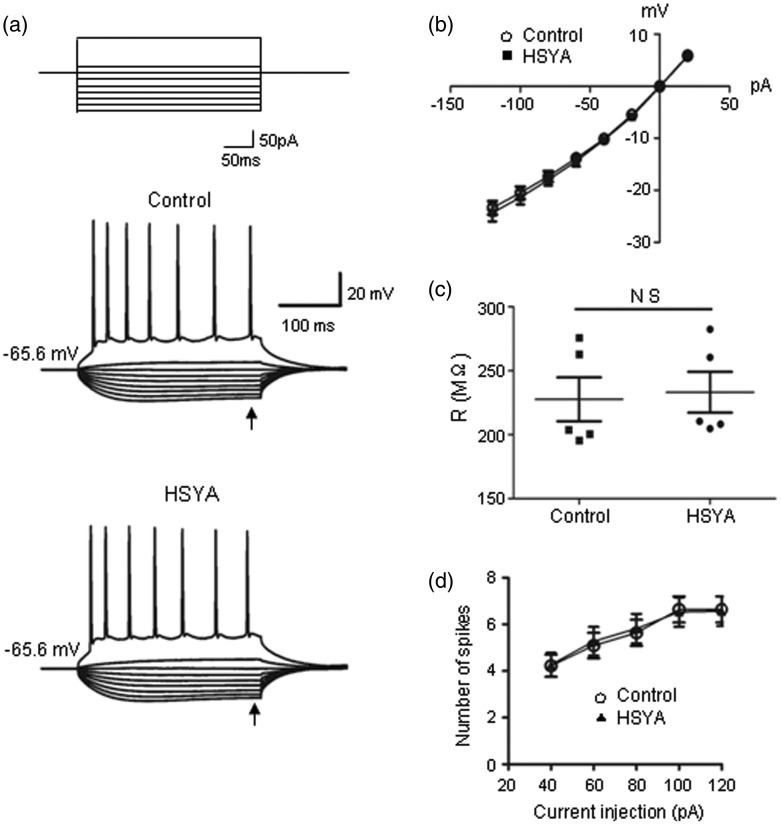
HSYA does not change NMDAR-related cell membrane property. (a) Representative traces showing the voltage responses of CA1 PCs to a series of current injections (top; currents of −120 pA to +20 pA with step increase of 20 pA, and also at +100 pA) in the absence (middle) or presence (bottom) of HSYA (100 μM). (b) I–V relation did not show any difference before and after HSYA application. (c) Scatter plot of input resistance derived from recordings shown in (a). The cell membrane resistances did not show significant change in the absence or presence of HSYA (*n* = 5; *p* = .14, paired *t* test). (d) The number of action potential was not altered by HSYA (*n* = 9; *p* = .40, paired *t* test). HSYA = hydroxysafflor yellow A; NMDAR = N-methyl d-aspartate receptor; PC = pyramidal cell.

Next, we examined HSYA's effect on the intrinsic properties of CA1 PCs in current-clamp mode by injecting stepped hyperpolarization and depolarization currents into neurons (see Methods section). We did not observe any obvious changes in the passive membrane property of neurons as related to NMDARs after bath application of HSYA (Figure 5a–c; *p* = .13, paired *t* test). We also evaluated whether the active membrane property of neurons was affected by HSYA application through injecting depolarization currents and measuring the resulting action potentials. We did not detect any changes in the firing frequency of the action potential after HSYA application (Figure 5a and d; *p* = .40, paired *t* test). These results suggest that although HSYA inhibits synaptic NMDAR activation, it does not alter the passive and active membrane properties of CA1 neurons.

### HSYA Suppresses NMDAR-Mediated OGD-Evoked Membrane Depolarization Current

It has been reported that neurons exposed to OGD for about 6 min exhibited an irreversible membrane depolarization (Centonze et al., 2001). This depolarization persisted even after removal of the OGD stimulus (Higashi et al., 1990; Allen et al., 2005; Murai et al., 2012).

In our experiments, we used an OGD solution to stimulate brain slices for 6 min, then returned the slices to the control solution. The NMDAR-mediated inward currents were continuously recorded before, during, and after OGD exposure.

**Figure 6.**
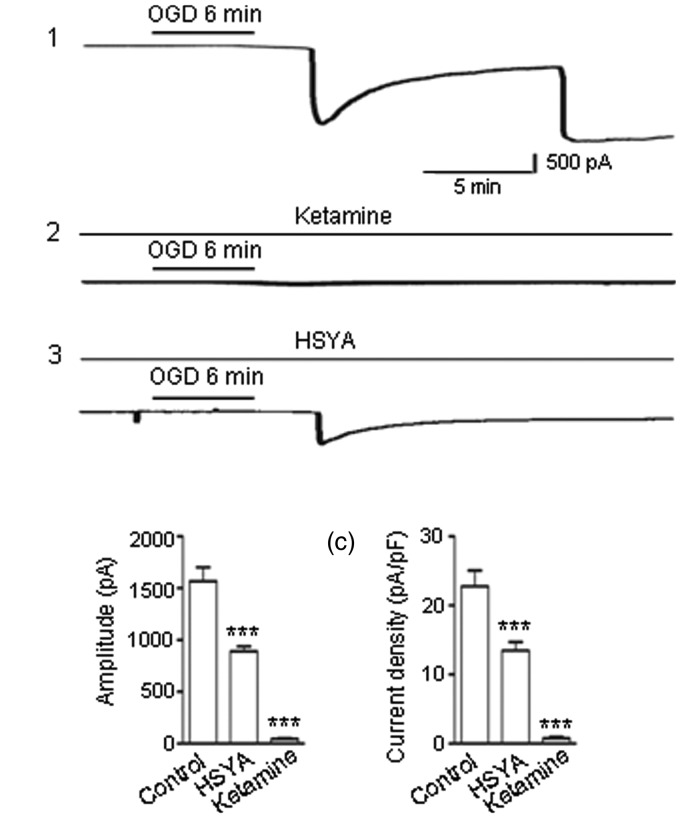
HSYA inhibits OGD-evoked and NMDAR-mediated depolarization current. (a) A 30-min OGD paradigm was applied, including a 5-min baseline in low Mg^2+^ ACSF containing CNQX (5 μM), strychnine (1 μM) and PTX (100 μM), followed by a 6 min OGD exposure (see Methods section), and finally by reperfusion of the slice with the control solution. OGD induced a large and persistent NMDAR-mediated membrane depolarization current (a1). When ketamine (30 μM) was included in the perfusion solution, the size of the OGD-evoked depolarization current was greatly reduced (a2). When HSYA (100 μM) was applied, the size of the OGD-evoked depolarization current was also markedly reduced (a3). (b) Bar graph showing OGD-evoked depolarization currents (*n* = 14). The amplitudes of the depolarization currents were greatly reduced by Ketamine (*n* = 6), and by HSYA (*n* = 7). (c) The current density (pA/pF), which indicates the charge transferred through NMDARs, was measured. Bar graph showing the mean current density (±*SE*). ***p < .001 compared with control, One-way analysis of variance. CNQX = 6-cyano-7-nitroquinoxaline-2,3-dione; HSYA = hydroxysafflor yellow A; NMDAR = N-methyl d-aspartate receptor; OGD = oxygen-glucose deprivation.

We observed a huge inward current occurring in neurons after 6 min of OGD exposure (Figure 6a), suggesting cell membrane depolarization. The inward current did not recover after the slices were reperfused in the control solution (Figure 6a1). Instead, the depolarizing current continued to increase in amplitude and persisted for the remainder of the recording. The mean amplitude of OGD-evoked peak currents was 1,569 ± 133 pA (Figure 6a1 and b). When brain slices were pretreated with NMDAR blocker ketamine (30 μM), OGD only induced a very small inward current (Figure 6a2 and b; 45 ± 12 pA), which confirmed the probability that the large inward current induced by OGD was NMDAR mediated. As HSYA inhibits NMDAR activity, we predicted that HSYA would also be able to suppress the OGD-induced, NMDAR-mediated lethal membrane depolarization. When brain slices were preincubated with HSYA prior to OGD exposure, we found that the amplitude of the OGD-induced NMDAR-mediated current was dramatically reduced to 890 ± 47 pA (Figure 6a3 and b; *p* < .001, one-way ANOVA).

We proceeded to measure the membrane current density (pA/pF) changes in the experiments described earlier and found that the changes in current density were parallel to the changes in peak current amplitude (Figure 6c; *p* < .001, one-way ANOVA): OGD caused a large current transfer through the unit membrane, while HSYA markedly suppressed this current transfer.

These results are consistent with the hypothesis that HSYA suppresses NMDAR-mediated neuronal membrane depolarization in OGD conditions, and affirm the possibility that HSYA exerts a neuroprotective effect in cerebral ischemia.

### HSYA Suppresses NMDAR-Dependent i-LTP

**Figure 7.**
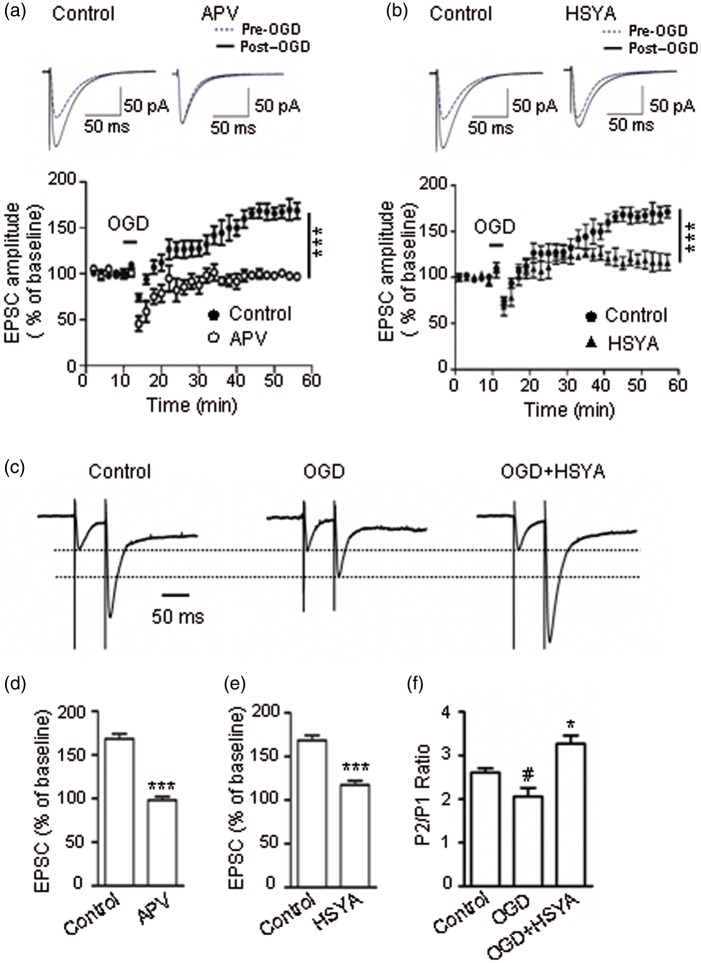
HSYA attenuates NMDAR-dependent i-LTP. (a) Evoked EPSCs following the electrical stimulation of Shaffer collateral fibers in mouse hippocampal slices. OGD exposure caused an increase in EPSC amplitudes; this potentiation effect lasted for more than 40 min (phenomenon known as i-LTP). When brain slices were preincubated with APV (100 μM), OGD could not induce an i-LTP. Left insert shows an increase in EPSC amplitude after OGD stimulation; right insert shows that APV abolished the increase in EPSC amplitude after OGD exposure. (b) The size of the NMDAR-dependent i-LTP was markedly suppressed when the slices were preincubated with HSYA (100 μM). Left insert shows that OGD induced an increase in EPSC amplitude; right insert shows that HSYA markedly attenuated the amplitude of EPSCs. (c) Normalized traces of AMPAR EPSCs evoked by paired-pulse stimulation. At the PP stimulus interval of 50 ms, there is a distinct increase in the amplitude of *P*2 compared with *P*1 under control conditions (left panel). OGD exposure markedly reduced the magnitude of enhancement of *P*2's amplitude (middle panel). However, in the presence of HSYA (100 μM), OGD exposure could not reduce the increase in *P*2's amplitude, but actually further enhanced it (right panel). (d–e) Bar graphs showing EPSC amplitudes as mean percentage (± *SE*) of control. APV (100 μM) completely abolished the NMDAR-dependent i-LTP current (c, *n* = 6), and HSYA (100 μM) markedly attenuated the magnitude of i-LTP (d, *n* = 6). ***p < .001 compared with control, unpaired *t* test. (f) Bar graphs showing the mean (± *SE*) *P*2/*P*1 ratio of (c). ^#^*p* < .05 compared with control group; *p < .05 compared with OGD group; paired *t* test. AMPAR = α-amino-3-hydroxy-5-methyl-4-isoxazolepropionic acid receptor; APV = 2-amino-5-phosphonopentanoic acid; HSYA = hydroxysafflor yellow A; i-LTP = ischemic long-term potentiation; NMDAR = N-methyl d-aspartate receptor; OGD = oxygen-glucose deprivation.

Long-term synaptic potentiation (LTP) in the CA1 region is NMDAR dependent; it plays very important roles in normal physiological functions such as neuronal development, learning, and memory. However, in pathological conditions such as brain ischemia, neurons undergo hypoxic injury and postsynaptic NDMAR overactivation, which results in the i-LTPs (Peineau et al., 2007). Due to its inhibitory effect on NMDARs, we predicted that HSYA would be able to suppress i-LTP and prevent excitatory neuronal death. To test this idea, CA1 neurons from hippocampal slices were voltage-clamped at −65 mV and perfused with low Mg^2+^ ACSF containing PTX and strychnine (see Methods section). After OGD insult, the amplitude of EPSCs was markedly increased to 168.60 ± 7.90% of the control baseline, and remained elevated for more than 40 min (Figure 7a). The size of the i-LTP current was reduced to 98.30 ± 3.90 % of the baseline when the slices were preincubated with NMDAR antagonist APV (100 μM; Figure 7a and d; *p* = .0008, unpaired *t* test). These data indicate that the OGD-induced i-LTP in the CA1 region was NMDAR dependent. The i-LTP current was suppressed to 118 ± 9 % of the baseline by pretreatment of slices with HSYA (Figure 7b and e; *p* = .0003, unpaired *t* test). These results confirm that HSYA is able to inhibit i-LTP and protect neurons from excitatory death through suppressing the overactivation of NMDARs.

We applied paired-pulse stimulation to examine HSYA's effect on presynaptic glutamate transmitter release in the OGD condition by recording AMPAR-mediated paired responses. At the stimulus interval of 50 ms, we found that in normal ACSF, the amplitude of the second AMPAR EPSC (*P*2) was facilitated compared with the first EPSC (*P*1), resulting in a PPR of 2.61 ± 0.08. The PPR was markedly reduced after OGD exposure (2.10 ± 0.13; *p*= .02; paired *t* test; Figure 7c and f). After OGD washout, the slices were incubated with HSYA (100 uM) and then reexposed to OGD. We found that the PPR was dramatically increased to 3.27 ± 0.16 (*p* = .02, paired *t* test). These data suggest that OGD exposure increases pre-synaptic glutamate transmitter release, and that HSYA is able to suppress this OGD-evoked pre-synaptic glutamate release.

### HSYA Prevents Ca^2+^ Entering Neurons Through NMDAR Channels

**Figure 8.**
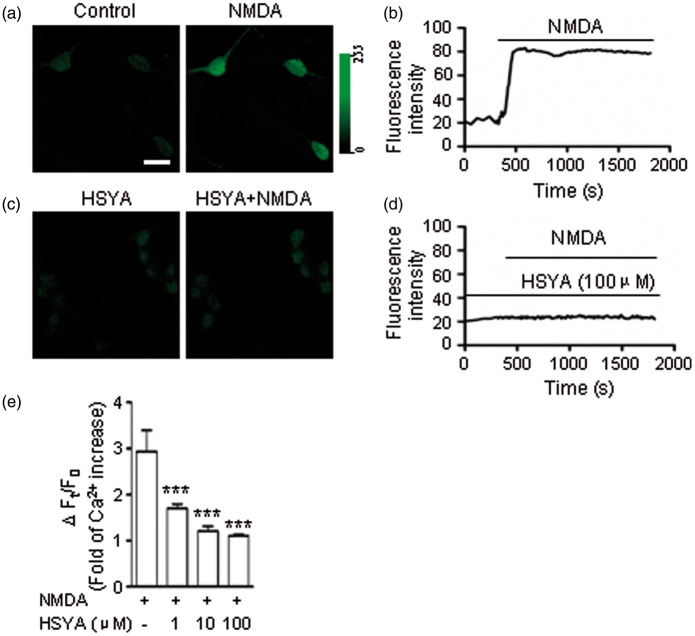
HSYA reduces Ca^2+^ influx through NMDARs. (a) Representative confocal images of cultured hippocampal neurons under the blockade of AMPAR, GABA_A_ receptors, glycine receptors, and VGCCs, respectively. NMDA induced an apparent increase in the intensity of green fluorescence (right panel) of cells compared to control (left panel). Scale bar is 15 µm. (b) Trace showing the changes in fluorescence intensity over time in representative neurons during the experiment. After NMDA treatment, there is a persistent increase in fluorescence intensity, indicating [Ca^2+^]_i_ increase. (c) When neuronal cultures were pretreated with HSYA (100 μM) (left panel), NMDA could not induce an increase in cell fluorescence intensity (right panel). (d) Trace showing that the neuronal fluorescence intensity was not increased following NMDA exposure when cultures were pretreated with HSYA (100 μM). (e) HSYA inhibited the [Ca^2+^]_i_ increase in hippocampal neurons in a concentration-dependent manner. Statistical analysis of the NMDA-evoked changes in neuronal fluorescence intensity (which reflects [Ca^2+^]_i_ changes) when cultures were pretreated with different concentrations of HSYA (1 μM, *n* = 16 neurons; 10 μM, *n* = 19 neurons; 100 μM, *n* = 22 neurons). HSYA (100 μM) almost completely inhibited the [Ca^2+^]_i_ increase. Bar graph showing the relative decrease in mean fluorescence intensity (± *SE*) of NMDA with HSYA (1, 10, and 100 μM, respectively) treated group compared with only NMDA-treated group. ***p < .001, One-way analysis of variance. AMPAR = α-amino-3-hydroxy-5-methyl-4-isoxazolepropionic acid receptor; APV = 2-amino-5-phosphonopentanoic acid; [Ca^2+^]_I_ = intracellular calcium concentration; GABA_A_ = γ-aminobutyric acid A-type; HSYA = hydroxysafflor yellow A; i-LTP = ischemic long-term potentiation; NMDAR = N-methyl d-aspartate receptor; OGD = oxygen-glucose deprivation; VGCC = voltage-gated calcium channel.

NMDAR overactivation by OGD is the major cause of excitatory cell death (Tymianski et al., 1993). It has been reported that the NMDAR-mediated Ca^2+^ influx into neurons is the main mechanism responsible for neuronal death (Sattler et al., 1998; Lau and Tymianski, 2010). To explore whether HSYA is able to inhibit Ca^2+^ entry into neurons through the suppression of NMDARs, we conducted Ca^2+^ imaging experiments in cultured hippocampal neurons (see Methods section). The intensity of cell fluorescence, which correlates with the intracellular calcium concentration ([Ca^2+^]_i_) in the neuron, was dramatically increased to 2.9 times that of control after NMDA application in the ECS (Figure 8a, b, and e; *n* = 16 neurons; *p* < .0001, one-way ANOVA). NMDAR blocker ketamine effectively prevented Ca^2+^ from entering into neurons (Gao et al., 2015). When neuronal cultures were pretreated with HSYA (100 μM), the NMDA-mediated [Ca^2+^]_i_ elevation was also inhibited (Figure 8c and d). In addition, we found that HSYA prevented the [Ca^2+^]_i_ increase in a concentration-dependent manner in the testing range of 1 to 10 μM (Figure 8e). The data derived from calcium imaging are consistent with the results of our electrophysiology experiments described earlier.

### HSYA Protects Hippocampal Neurons From NMDAR-Induced Cell Death

**Figure 9.**
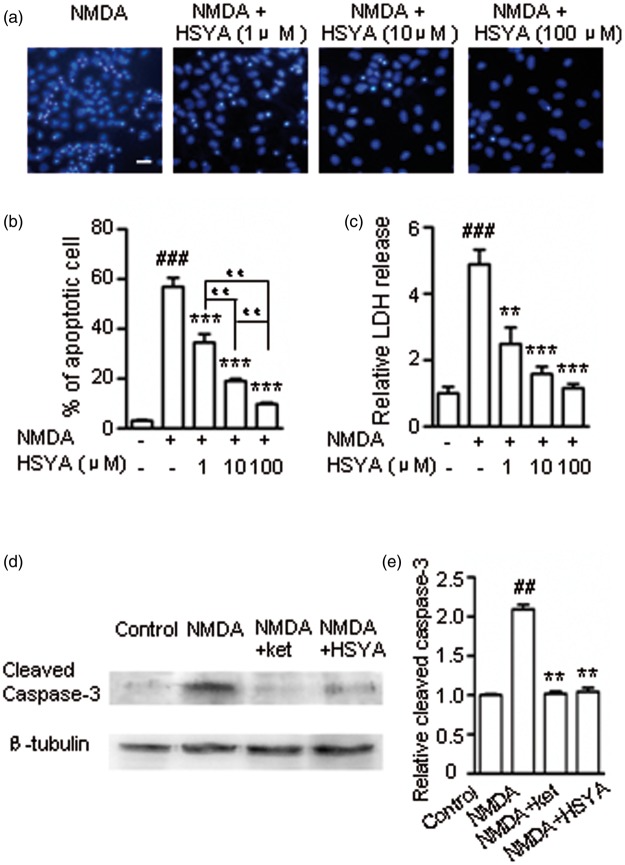
HSYA protects hippocampal neurons from NMDA-induced cell death. (a) Cultured hippocampal neurons were stained with Hoechst-33342. NMDA (100 μM) and glycine (10 μM) treatment induced typical apoptotic cell death. When cultures were pretreated with 1 μM, 10 μM, and 100 μM of HSYA, respectively, the number of apoptotic neurons were all reduced. Scale bar is 20 µm. (b) Statistical analysis of the protective effect of HSYA on neurons, as shown in the bar graph. With increasing concentrations of HSYA (from 1 μM to 100 μM), the apoptotic cell death rates were reduced to 34.50 ± 4.50 % (*n* = 5), 19 ± 0.70 % (*n* = 5), and 9.70 ± 0.90 % (*n* = 5), respectively. Bar graph showing the mean death rate (± S.E.) of (a). ^###^*p* < .001 compared with the control group. ***p < .001 compared with the NMDA treatment group; ^# #^*p*< .01 compared in different concentrations of HSYA group. One-way ANOVA. (c) The relative level of LDH release in the culture medium was measured and shown in the bar graph. Bar graph showing the mean relative LDH release (± *SE*). ^###^*p* < .001 compared to the control group, **p < .01 and ***p < .001, respectively, compared with the NMDA treatment group, One-way ANOVA. (d) Western blot data showing the level of cleaved caspase-3 production (activated caspase-3, ∼17 KD) in different treatment conditions. (e) The protein band density from (d) is shown in the bar graph. NMDA treatment of the hippocampal cultures induced a 2.2-fold increase in production of cleaved caspase-3 (*n* = 4) compared with control; ketamine reduced the NMDA-induced cleaved caspase-3 production to 1.03-fold that of control (*n* = 4); HSYA also significantly reduced NMDA-induced cleaved caspase-3 production to 1.04-fold that of control (*n* = 4). Bar graph showing the mean relative density (± *SE*) of control of (d). ^##^*p* < .01 compared with the control group; **p < .01 compared with the NMDA group. One-way ANOVA. ANOVA = analysis of variance; NMDA = N-methyl d-aspartate receptor. LDH = lactate dehydrogenase; NMDA = N-methyl d-aspartate.

To verify that HSYA is able to protect neurons from apoptotic death caused by the overactivation of NMDARs, we conducted cell viability assays. Using Hoechest-33342 staining, apoptotic neurons are visualized as cells displaying the characteristic morphological changes of cell shrinkage, nuclear condensation, and fragmentation (Figure 9a). We observed that NMDA treatment of hippocampal cultures induced a 55.20 ± 3.70% neuronal death rate. The increase in apoptotic cell death was prevented by pretreatment with HSYA (100 μM). The protective efficacy of HSYA was positively correlated with the concentration of HSYA (1–100 μM; Figure 9a and b; control cultures: 3.11 ± 0.46%, *p* < .001, one-way ANOVA). In addition, HSYA at its highest concentration (100 μM) did not have any observable adverse effect on cultures (data not shown).

LDH release into the extracellular environment has been a useful indicator of cell necrosis (Xiong et al., 2004). NMDA treatment of hippocampal cultures caused a 2.48 ± 0.49 fold increase in LDH concentration in the ECS (Figure 9c). This NMDA-induced LDH increase was effectively prevented by pretreatment of cultures with HSYA (100 μM). Again, the protective efficacy of HSYA was positively correlated with the concentration of HSYA (1–100 μM). Thus, HSYA appears to be able to inhibit both cell apoptosis and necrosis induced by NMDA. These results provide further support for the hypothesis that HSYA protects neurons against NMDAR-mediated excitotoxic death.

Taking an additional step, we measured the production level of cleaved caspase-3, one of the main effector enzymes that carry out the cell death program (D'Amelio et al., 2012). Our data showed that NMDA treatment of neuronal cultures caused a greater increase in the production of cleaved caspase-3 compared with control (Figure 9d and e). This increase was markedly reduced by preincubation of the cultures with either NMDAR antagonist ketamine or HSYA (Figure 9d and e; *p* < .01, one-way ANOVA). This finding is consistent with the results of our calcium imaging and cell viability assays, and provides additional supportive evidence for the neuroprotective effect of HSYA.

### HSYA Protects Mitochondria From NMDA-Induced Damage

**Figure 10.**
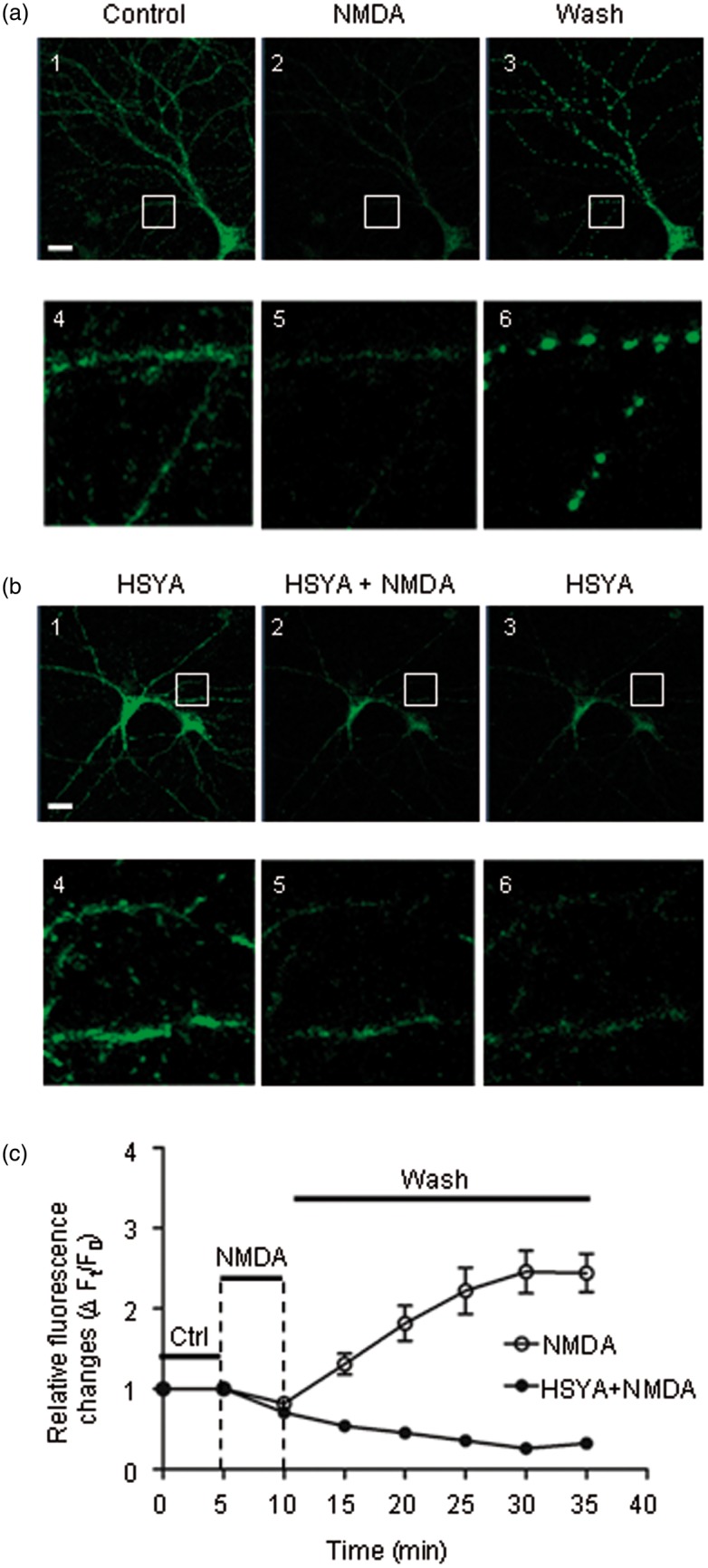
HSYA prevents mitochondria from NMDA-induced damage. (a) Representative confocal images showing the cultured hippocampal neurons transfected with mt-cpYFP plasmid under different treatments. Bottom row (images 4–6) shows enlarged images of the areas marked with a white box in the top row (images 1–3). The fluorescence allows visualization of the distribution and morphology of neuronal mitochondria. The control image is shown in the left panel. After NMDA treatment, the fluorescence intensity of the mitochondria was greatly reduced (middle panel). The fluorescence intensity was dramatically increased after removal of NMDA from the perfusion solution, and the mitochondria showed fragmented and punctiform changes in morphology (right panel). Scale bar is 20 µm. (b) Cultures were pretreated with HSYA (20 μM) prior to adding NMDA in the perfusion solution (left panel). After NMDA exposure, the reduction in mitochondrial fluorescence intensity with HSYA pretreatment was much less compared to that of NMDA treatment without HSYA (middle panel). In addition, with HSYA treatment, the fluorescence intensity was not increased after removal of NMDA from perfusion solution. Neither fragmented nor punctiform mitochondria were seen (right panel). Bottom row (images 4–6) shows enlarged images of the areas marked with a white box in the top row (images 1–3). (c) Quantitative analysis of relative mitochondrial fluorescence intensity with and without HSYA pre-treatment. After NMDA exposure, the fluorescence intensity went down for about 5 min and then started to rise up rapidly (open circle), until eventually reaching a plateau around 30 min. When the culture was pretreated with HSYA (20 μM), NMDA induced a reduction in the fluorescence intensity; after wash, the fluorescence intensity did not rise again. HSYA = hydroxysafflor yellow A; mt-cpYFP = mitochondrial-targeted circularly permuted yellow fluorescent protein; NMDA = N-methyl d-aspartate.

Biosensor mt-cpYFP was used as a marker for mitochondrial matrix alkalization, reactive oxygen species (ROS) production, mitochondrial membrane potential disruption, and mitochondrial morphology change (Cheng et al., 2014). Using a confocal microscope under low magnification, the mitochondria-expressed mt-cpYFP was seen to be distributed throughout neuronal soma and dendrites under control conditions (Figure 10a1). Mitochondria were identified by their small, bright, and round- or rod-shaped structures under high magnification (Figure 10a4). When neuronal cultures were perfused in NMDA-containing solution, mt-cpYFP fluorescence intensity decreased rapidly (Figure 10a2, a5, and c). However, after removing NMDA from the perfusion solution, the fluorescence intensity recovered for a short period of time before increasing to almost 3 times the intensity of control (Figure 10c). Neuronal mitochondria also displayed increasingly fragmented and punctiform morphology (Figure 10a3 and a6). The fluorescence intensity reduction suggests that mitochondria were undergoing swelling due to the dilution of the mt-cpYFP within the mitochondrial matrix. The fragmentation and punctiform appearance suggest that the mitochondria were broken down as ROS production increased, and as mitochondrial membrane potential and ion homeostasis were disrupted.

When neuronal cultures were preincubated with HSYA (20–30 μM), NMDA treatment induced only a slight reduction in mt-cpFYP fluorescence intensity (Figure 10b1–b2 and b4–b5), suggesting that there is less mitochondrial swelling. After removing NMDA from perfusion solution, the fluorescence intensity remained low, and the mitochondria did not exhibit fragmented or punctiform appearance. (Figure 10b3 and b6). These data suggest that HSYA protects neurons from excito-toxic damage and death through stabilizing mitochondrial structures. This is consistent with the results of the Ca^2+^ imaging and Western blot experiments.

## Discussion

It is well known that NMDAR is an ion channel with high Ca^2+^ permeability, which allows it to mediate Ca^2+^ influx during channel activation. Ca^2+^, as the second messenger, plays a pivotal role in many physiological conditions including cell growth, neurodevelopment, learning, memory, and synaptic plasticity. However, in pathological conditions, overactivation of NMDARs can cause large amounts of Ca^2+^ influx into neurons, triggering cellular damage or death. Due to the shortage of adenosine triphosphate, Ca^2+^ accumulated within the cell activates many Ca^2+^-dependent enzymes, causing damage to protein, lipid, and DNA (Coyle and Puttfarcken, 1993; Simonian and Coyle, 1996; Lee et al., 1999). The Ca^2+^ signaling pathway can also cross talk with the PI3K/Akt/GSK3β signaling pathway, resulting in neuronal damage (Jiang et al., 2015).

Previous studies have shown that the NMDAR-mediated Ca^2+^ influx activates NF-κB, increases pro-inflammatory cytokine production (IL-1β, IL-6, and TNF-α), and exacerbates NMDAR-mediated excitatory toxicity (Jander et al., 2000; Kawasaki et al., 2008; Gao et al., 2015). Others studies have demonstrated that only Ca^2+^ entering the neuron through NMDARs can cause the calcium-mediated neurotoxicity (Sattler et al., 1998; Lau and Tymianski, 2010). The explanation given is that through this pathway, Ca^2+^ triggers the formation of the NMDA–nNOS–PSD-95 complex beneath the internal surface of the cell membrane, and thus easily activates neuronal nitric oxide synthase (nNOS; Sattler et al., 1999). When nNOS is activated, it produces more NO gas (Sattler et al., 1999). NO is able to diffuse into pre-synaptic terminals, causing the activation of guanylyl cyclase and increased pre-synaptic cyclic guanosine monophosphate production, which leads to increased pre-synaptic transmitter release (Costa et al., 2011). NO can also freely diffuse into the cytosol and react with the free radical superoxide to form peroxynitrite, a potent oxidant that can cause protein nitration/oxidation, lipid peroxidation, and direct DNA damage (Radi et al., 1991a, b).

In our experiments we have shown that HSYA inhibited NMDARs and suppressed pre-synaptic transmitter release in the CA1 region of mouse hippocampus. The underlying mechanism of its action might be the inhibition of NMDA-PSD95-nNOS complex-dependent NO production. This idea was supported by the experimental results that HSYA reduced postsynaptic NMDAR EPSCs and increased PPR of both NMDAR EPSCs and AMPAR EPSCs.

HSYA is found to exert a distinct neuroprotective effect and stabilize the mitochondrial membrane, as manifested by its ability to reduce neuronal membrane depolarization, intracellular Ca^2+^ influx, cleaved caspase-3 production, LDH generation, nNOS activation, and ROS generation in the mt-cpYFP–labeled mitochondria. These experimental results point to some important underlying themes: (a) NMDAR-mediated intracellular Ca^2+^ overload is the primary mechanism for the pathological changes in brain ischemia, and (b) HSYA, through inhibiting the NMDAR-mediated Ca^2+^ influx, can prevent all of these abnormal cellular changes.

OGD exposure mimics brain ischemia and triggers neuronal membrane depolarization, NMDAR activation, and Ca^2+^ influx (Crepel et al., 2003). After brain ischemia, neurons in the ischemic core region die rapidly, but the penumbral region also undergoes short-term energy shortage and pathological excitation, including development of the i-LTP. In this study, we found that HSYA effectively suppressed the i-LTP induced by exposure to OGD. The inhibitory power of HSYA on i-LTP was not as great as that of NMDAR-selective antagonist APV. This makes HSYA a potentially ideal agent for the treatment of ischemic brain damage because the brain needs to maintain an adequate level of NMDAR activity in order to preserve physiological synaptic function and promote neuronal recovery after ischemia. This theory can well explain why HSYA has been shown to reduce infarct volume in ischemic animal models.

HSYA did not exhibit any selective inhibitory effect on either the NR2A or NR2B subunit. We can thus infer that the binding site of HSYA on NMDARs probably lies somewhere on the NR1 subunit. More experiments need to be done in the future to examine this hypothesis.

As HSYA is a water-soluble molecule, it cannot easily cross the neuronal lipid bilayer membrane. Although it has been reported that HSYA causes a reduction in nitrotyrosine production, and displays antioxidant and anti-inflammatory effects, the detailed mechanism of its action remains unclear. We demonstrate for the first time that HSYA inhibits NMDARs at the neuronal membrane and prevents ischemic cell damage from a point upstream of cellular death cascades. We predict that HSYA may become a promising agent in the treatment of cerebral ischemia.
